# Urinary tract infection in the neonatal intensive care unit

**DOI:** 10.1038/s41372-026-02690-1

**Published:** 2026-04-29

**Authors:** Jacqueline Magers, Alexandra Burton, Pavel Prusakov, Natalie O. White, Randy R. Miller, Richard Moraille, Anthony R. Theile, Pablo J. Sánchez

**Affiliations:** 1 Department of Pharmacy, Nationwide Children’s Hospital, Columbus, OH USA; 2 Pediatrix Medical Group of Ohio, Columbus, OH USA; 3 Central Ohio Newborn Medicine, Columbus, OH USA; 4 Department of Pediatrics, Divisions of Neonatology and Pediatric Infectious Diseases, Nationwide Children’s Hospital, Abigail Wexner Research Institute at Nationwide Children’s Hospital, Center for Perinatal Research, The Ohio Perinatal Research Network, The Ohio State University College of Medicine, Columbus, OH USA; 5 Department of Pediatrics, Division of Neonatology, Nationwide Children’s Hospital, Center for Perinatal Research, Abigail Wexner Research Institute at Nationwide Children’s Hospital, The Ohio State University College of Medicine, Columbus, OH USA; 6 Department of Pediatrics, Division of Pediatric Infectious Diseases, Nationwide Children’s Hospital, The Ohio State University College of Medicine, Columbus, OH USA; 7 Department of Neonatal Nurse Practitioners, Nationwide Children’s Hospital, The Ohio State Wexner Medical Center, Columbus, OH USA

**Keywords:** Bacterial infection, Medical research

## Abstract

**Objective:**

To determine adherence and safety of a diagnostic (culture, urinalysis) and therapeutic (5-day antibiotic course with a time-out) guideline for urinary tract infection (UTI) in the Neonatal Intensive Care Unit

**Study design:**

Prospective surveillance of non-bacteremic UTIs from 11/2020-12/2022. Safety outcomes were defined by re-initiation of antibiotics within 7 days for a subsequent UTI due to the same organism and overall and UTI-related mortality.

**Results:**

77 infants received treatment for 93 UTIs with 77% (72/93) adherence to diagnostic criteria; 90% (82/91) of infants received ≤6 days of definitive treatment (median [IQR] antibiotic duration 5 [5–6] days). Antibiotics were restarted within 7 days for a recurrent (same organism) UTI in 1/91 (1%) UTIs. Mortality was 4% (4/93); none were due to UTI.

**Conclusion:**

Adherence to diagnostic UTI criteria was 77%. 90% of infants received short course treatment that was associated with a 1% failure rate. No safety concern was identified.

## Introduction

Urinary tract infection (UTI) is the most frequent serious bacterial infection in early infancy, occurring in 4.6% to 13.6% of infants [1–6]. The prevalence among preterm and high-risk neonates in the neonatal intensive care (NICU) has been estimated to be even higher, with up to 25% of premature infants diagnosed with a UTI [7]. At the same time, there is no guidance for the management of UTI among infants in the NICU. The American Academy of Pediatrics (AAP) published guidelines for the diagnosis and treatment of UTIs in febrile infants aged 2–24 months [8]. Aspects of the guidelines could be applicable to infants <2 months of age, but additional studies examining how to best apply these guidelines to the neonatal population are needed [6, 9, 10]. Specifically, studies evaluating diagnostic criteria and duration of therapy are warranted [11].

In August 2019, the Neonatal Antimicrobial Stewardship Program (NEO-ASP) at Nationwide Children’s Hospital, Columbus, OH, and its seven affiliated NICUs implemented a diagnostic and therapeutic guideline that recommended performance of urinalysis for pyuria in addition to bacterial culture and a 5-day antibiotic course with a time-out for treatment of UTI in infants without concomitant bacteremia or meningitis (Table 1). The objective of this study was to determine the adherence to the diagnostic and therapeutic aspects of the guideline and its safety outcomes among NICU infants.

**Table 1:** Guideline for diagnosis and management of urinary tract infection (UTI) in the neonatal intensive care unit.

**1**. All infants undergoing a sepsis evaluation *after 3 days of age* should have a urine analysis (including urine NGAL [neutrophil gelatinase-associated lipocalin] if available) and culture performed.
**2**. Urine sample should be obtained by catheterization before antibiotic initiation (suprapubic bladder aspiration [SBA] is an alternative^a^).
3. Criteria for treatment:
a. ≥ 50,000 CFU/mL (either I&O [In and Out] catheterization or SBA), with or without pyuria (≥5 WBC/HPF [centrifuged urine] or ≥10 WBC/mm^3^ [unspun])
b. Lower colony count (≥10,000) may be treated if associated with pyuria
c. Polymicrobial urine culture: consider as contaminant unless repeated and shows significant colony count of single organism as indicated above^3a,3b^
4. Treatment should be guided by culture results. Consider the following:
a. High suspicion for UTI: gentamicin and ampicillin (provides *Enterococcus* coverage)
b. General late-onset sepsis (> 3 days of life) workup: gentamicin and nafcillin (does not provide enterococcal coverage; if UTI suspected, treat with ampicillin/sulbactam instead of nafcillin)
c. General late-onset sepsis (> 3 days of life) workup in a patient with *MRSA* colonization/past infection: gentamicin and vancomycin
d. Base definitive treatment on culture and susceptibility results
5. Treat for 5 days for UTI without bacteremia. A “TIME-OUT” should be performed on day #5 of treatment to see if longer duration (i.e., 7 days) is warranted or other diagnoses possible.
6. No follow-up urine culture needed, either at 24–48 h after initiation of antibiotics or after completion of therapy, unless:
a. Bacterial isolate is multi-drug resistant (e.g., ESBL Gram-negative bacilli)
b. Lack of clinical response
c. VCUG (voiding cystourethrogram) is performed (see below #8)
7. Renal ultrasound (RUS) should be performed during the course of antibiotic therapy.
8. VCUG should be performed only after a 2nd UTI or if the RUS is abnormal for renal disease or significant reflux (e.g., hydronephrosis, scarring, findings suggestive of high-grade vesicoureteral reflux, or obstructive uropathy) and in consultation with nephrology/urology. Perform a urine culture before performance of VCUG to document sterilization.
9. Infants should not be prescribed antibiotic prophylaxis.

*WBC* white blood cell, *HPF* high power field, *MRSA* methicillin-resistant *Staphylococcus aureus*, *ESBL* extended spectrum beta-lactamase.

^a^If suprapubic aspiration is unsuccessful x 1, an I&O catheterization should be performed before starting antibiotics. Contraindications to suprapubic tap may include: bleeding diathesis, abdominal distension, massive hepatosplenomegaly, overlying cellulitis, lower abdominal wound or scar, bladder anomaly or tumor, deemed unstable for procedure.

## Methods

This prospective study included all infants who received antibiotic therapy for a diagnosis of UTI at 7 Nationwide Children’s Hospital NICUs (Level 4: *n* = 1, outborn; Level 3: *n* = 5, inborn; Level 2: *n* = 1, inborn; 263 total beds) from November 1, 2020, to December 31, 2022. Infants were excluded if they had concurrent bacteremia or meningitis, although the lack of performance of a blood culture or lumbar puncture was not an exclusion criterion. Infants also were excluded if the if the urine culture yielded fungal growth or the urine sample was obtained via a nephrostomy catheter. Infants were identified by NICU pharmacists during daily (Monday to Friday) patient rounds as well as during twice-weekly NEO-ASP meetings, where all infants on antibiotic therapy were discussed and recommendations provided to the attending neonatologist or advanced practice providers in real time. Recommendations were relayed via the electronic health record’s secure chat function or by phone or Vocera Badge (Vocera Communications, San Jose, CA). Following the NEO-ASP recommendation for a 5-day antibiotic treatment course for non-bacteremic UTI (Table 1), NICU pharmacists monitored its implementation on patient rounds. Prospective demographic, clinical, laboratory, microbiologic, and radiologic data were obtained from the electronic health record and managed using REDCap (Research Electronic Data Capture) tool hosted at Nationwide Children’s Hospital [12, 13]. REDCap is a secure, web-based, software platform designed to support data capture for research studies, providing (1) an intuitive interface for validated data capture; (2) audit trails for tracking data manipulation and export procedures; (3) automated export procedures for seamless data downloads to common statistical packages; and (4) procedures for data integration and interoperability with external sources. The following information was compiled: urine collection method, results of urine analysis, urine culture results, antibiotic therapy, antibiotic restart within 7 days of completion of UTI treatment course, occurrence of a recurrent UTI, antibiotic prophylaxis following UTI, renal ultrasound and voiding cystourethrogram findings, and hospital outcome (discharge or death). Per the guideline, significant bacteriuria was defined as ≥ 50,000 colony forming units (CFU)/mL, with lower colony counts (≥10,000 CFU/mL) treated if associated with pyuria. Significant pyuria was defined as centrifuged urine having ≥5 WBC/high-power field or unspun urine with ≥10 WBC/mm^3^ (Table 1) [8].

Evaluation for possible UTI was performed among infants >3 days of age when possible late-onset sepsis was suspected. The components of the sepsis evaluation were at the discretion of the attending neonatologist. Per NEO-ASP guidance, empiric antibiotic therapy for suspected late-onset sepsis, defined as >3 days of age, was nafcillin and gentamicin in infants without a history of methicillin-resistant *Staphylococcus aureus* colonization or infection, irrespective of clinical or laboratory parameters such as the presence of a central venous catheter, hypotension, or abnormal acute phase reactants [14]. Infants who were known to be colonized with methicillin-resistant *S. aureus* at the time of the sepsis evaluation received empiric vancomycin (rather than nafcillin) in addition to gentamicin treatment. If urinary tract infection was strongly suspected, ampicillin was provided as an alternative agent to nafcillin. Subsequent antibiotic therapy was based on results of urine culture obtained as part of the sepsis evaluation.

In addition to the specific antibiotic prescribed, the start and stop dates for all antibiotics provided to infants who were diagnosed with UTI were recorded, including if provided intravenously or by the enteral route. The length of therapy was defined as any calendar day that the infant received ≥1 antibiotic for treatment of UTI. In infants <28 weeks’ gestation, one aminoglycoside (gentamicin or tobramycin) dose was considered as 2 days of antibiotic therapy, as the dosing of aminoglycosides for that age group was once every 48 h. Definitive antibiotic therapy was defined as the actual treatment course that was decided upon by the treatment team based upon clinical course, results of urine culture and urinalysis, and antibiotic susceptibility testing. Safety outcomes were defined a priori by re-initiation of antibiotic therapy within 7 days after discontinuation of the definitive treatment course for a subsequent UTI due to the same organism (recurrent infection) as well as overall and sepsis-related mortality while in the NICU. Subsequent episodes of positive blood or cerebrospinal fluid cultures but sterile urine culture among infants following a UTI were not included in the analysis, but all infants were followed through NICU discharge for mortality. In addition, adherence to the diagnostic and treatment aspects of the UTI guideline was assessed.

The study was approved by the Institutional Review Board of Nationwide Children’s Hospital with waiver of informed consent. The study was performed in accordance with the Declaration of Helsinki.

### Statistical analyses

Descriptive data were analyzed as median with interquartile range (IQR). Data management and descriptive statistics were conducted using Microsoft Excel.

## Results

During the 26-month study, 77 infants were diagnosed with 93 bacterial UTIs in the NICU. Two additional infants had urine cultures that yielded *Candida* species and were excluded from analysis. Of the 77 infants, the first UTI episode occurred at a median (IQR) age of 43 (25–72) days (Table 2). Their median (IQR) gestational age at birth was 30 (26–35) weeks, and birth weight was 1318 (736–2342) grams, with 66% (51/77) being male. Of the 77 infants, 62 (81%) met the diagnostic criteria recommended by the guideline (Table 2). Of those who did not meet the criteria for treatment (Table 1), 12% (9/77) of infants had <50,000 CFU/mL in the urine culture but no pyuria, while 6% (5/77) had <10,000 CFU/mL in the urine culture but had pyuria with a range of 8–136 WBC/high-power field in the urinalysis. One additional infant received treatment for UTI, but urine for culture was unable to be obtained. The majority of infants (45/77, 58%) had a normal renal ultrasound. Thirteen of the 77 infants (17%) had a voiding cystourethrogram performed, and 62% (8/13) were normal (Table 2). Abnormalities relating to vesicoureteral reflux were seen in the other 5 infants.

**Table 2:** Characteristics of the 77 infants who were diagnosed with a first episode of urinary tract infection.

	First urinary tract infection
No. of infants	77
Gestational age (week; median, IQR)	30 (26–35)
Birth weight (grams; median, IQR)	1318 (736–2342)
Chronologic age at diagnosis (day; median, IQR)	43 (25–72)
Male (*n*, %)	51 (66)
Urine bacterial colony-forming unit/ml, pyuria^a^:	
≥ 50,000	56 (73%)
10,000–49,000 + pyuria	6 (8%)
10,000–49,000 without pyuria	9 (12%)
<10,000 + pyuria	5 (6%)
No culture	1 (1%)
Diagnostic criteria met	62 (81%)
Renal ultrasound (*n* = 77):	
Normal	45 (58%)
Abnormal:	32 (42%)
Absent kidney	1
Cortical cysts	1
Dilated ureter	1
Duplex morphology	3
Echogenic kidneys	3
Horseshoe kidney	2
Mild pelviectasis	7
Hydronephrosis	7
Parenchymal renal disease	1
Multicystic dysplastic kidney	1
Mild urinary tract dilation (P1)	1
Nephrocalcinosis	6
Nephrolithiasis	4
Severe hydroureteronephrosis	1
VCUG (*n* = 13)	
Normal	8 (62%)
Abnormal:	5 (38%)
VUR Grade III	2 (3%)
VUR Grade IV	3 (4%)
Mortality	3 (4%)^b^

*IQR* interquartile range, *VCUG* voiding cystourethrogram, *VUR* vesicoureteral reflux.

^a^Pyuria definition: ≥5 WBC/HPF (centrifuged urine) or ≥10 WBC/mm^3^ (unspun).

^b^1, necrotizing enterocolitis (totalis); 1, hypoxic brain injury post cardiorespiratory failure; 1, partial midgut volvulus and compartment syndrome.

Of the 77 infants, 12 (16%) received treatment for >1 episode of UTI (Table 3). Nine infants had 1 subsequent UTI episode, 2 infants had 2 subsequent episodes, and 1 infant had 3 subsequent episodes for a total of 16 additional UTI episodes among the 12 infants. None of the subsequent UTIs were associated with bacteremia. Of the 16 episodes, 63% (10/16) met the diagnostic criteria of the UTI guideline. The median (IQR) time between UTI occurrences, that is, the number of days from the last day of antibiotic treatment for the UTI to the first day of the subsequent UTI treatment, was 21(8–44) days. The same organism that was cultured from the previous UTI was isolated in 9 (56%) of the 16 subsequent episodes (Table 4). Of the 12 infants, 4 (33%) had antibiotics re-initiated within 7 days after discontinuation of the initial definitive treatment course, but only 1 episode was due to the same organism (*Pseudomonas aeruginosa*) for an overall failure rate of 1% (1/91; Tables 3 and 4). None were receiving antibiotic prophylaxis at the time of the second UTI, but 2 infants received prophylaxis with amoxicillin after the second episode. Five (42%) of the 12 infants with recurrent UTIs had a normal voiding cystourethrogram, while 2 (17%) infants had vesicoureteral reflux (1, right grade III/left grade I; 1, right grade III). Five (42%) infants did not have a voiding cystourethrogram performed during the NICU stay.

**Table 3:** Characteristics of the 12 infants who were diagnosed with 16 subsequent episodes of urinary tract infection.

	Subsequent urinary tract infection
No. of infants	12
No. of episodes	16^a^
Gestational age (week; median, IQR)	29 (24–37)
Birth weight (grams; median, IQR)	1235 (760–1930)
Age (median, IQR)	84 (72–101)
Male (*n*, %)	9 (75%)
Urine bacterial colony-forming unit/ml, pyuria^b^:	
≥ 50,000	10
10,000–49,000 + pyuria	0
10,000–49,000 without pyuria	5
Urine unable to be obtained	1
Diagnostic criteria met (*n*, %)	10 (63%)
Interval between UTIs (days; median, IQR)	21 (8–44)
No. of infants whose subsequent UTI episode occurred ≤7 days from completion of antibiotic therapy	4 (33%)
Same pathogen as previous episode	1 (8%)^c^
Mortality	1 (8%)^d^

*IQR* interquartile range, *UTI* urinary tract infection.

^a^Nine infants had 1 subsequent UTI episode, 2 infants had 2 subsequent episodes, and 1 infant had 3 subsequent episodes for a total of 16 additional UTI episodes among the 12 infants.

^b^Pyuria definition: ≥5 WBC/HPF (centrifuged urine) or ≥10 WBC/mm^3^ (unspun).

^c^1 infant had same organism (1, *Pseudomonas aeruginosa*) isolated from subsequent UTI.

^d^Infant with atrioventricular canal, bronchopulmonary dysplasia, pulmonary hypertension.

**Table 4:** Characteristics of the 93 urinary tract infections diagnosed in 77 infants in the neonatal intensive care unit.

Total no. of antibiotic courses for urinary tract infection	93
Method of urine collection	
Catheterization	82 (88%)
In-dwelling Foley catheter	3 (3%)
Suprapubic bladder aspiration	0
Spontaneous void (“clean catch”)	5 (5%)
Bag (ureteral stents)	1 (1%)
Urine unable to be obtained	2 (2%)
No. of episodes that met diagnostic criteria	72 (77%)
Length of antibiotic therapy (days; median, IQR)	5 (5–6)
Test of cure performed	20/91^a^ (22%)
Antibiotics restarted ≤7 days from completion of UTI treatment (*n* = 12 infants)	14/91^a,b^ (15%)
Antibiotics restarted for subsequent UTI episode ≤7 days from completion of previous UTI treatment	4 (4%)
Same pathogen as previous episode	1/91^c^ (1%)
All subsequent UTI episode and pathogen:	
Same pathogen as previous UTI	9 (56%)
Different pathogen	6 (38%)
Urine unable to be obtained	1 (6%)
Days between UTI episodes in patients with recurrent infections^d^ (median; IQR)	21 (8–44)
Mortality	4 (4%)
Infection-related	0

^a^2 infants excluded since discharged home on antibiotic therapy.

^b^4, fever; 2, necrotizing enterocolitis; 2, apnea; 1, *Ureaplasma spp*. meningitis; 1, bilious emesis; 1, pneumonia; 1, hypothermia/lethargy; 1, cardiorespiratory arrest; 1, abnormal renal ultrasound.

^c^1, *Pseudomonas aeruginosa* isolated from second and subsequent UTI.

^d^Number of days from the last day of antibiotic treatment for the UTI to the first day of the subsequent UTI treatment.

Eighty-eight percent of all urine cultures were obtained by catheterization (82/93, Table 4). Overall adherence to the diagnostic criteria of the guideline was 77% (72/93). Table 5 lists the organisms isolated from urine cultures obtained from infants in whom the UTI did or did not meet diagnostic criteria. For both groups, the most frequent organisms were *Enterococcus faecalis* and *Escherichia coli*, followed by other Gram-negative bacilli. With respect to antibiotic therapy, 49% (46/93) of UTI episodes were treated intravenously, with 37% (34/93) changed to an enteral agent, most commonly on day 3 of therapy. Exclusively enteral antibiotic therapy was utilized in 14% (13/93) of UTI episodes. The median (IQR) length of antimicrobial therapy was 5 (5–6) days, irrespective of whether the antibiotic was administered intravenously or enterally. Of the 91 infants who completed treatment while in the NICU, 65/91 (71%) received 5 calendar days of treatment, while 15/91 (16%) received 6 days, and 2/91 (2%) received 4 days. The majority of the UTIs were treated with gentamicin (29/93; 31%) or ampicillin (14/93; 15%) (Table 5).

**Table 5:** Organisms detected in 91 urine cultures and antibiotic treatment provided to 77 infants diagnosed with urinary tract infection.

Organism	1st UTI (*n* = 77)	Recurrent UTIs (*n* = 16)	Total (*n* = 91)
UTI: met diagnostic criteria			
* Enterococcus faecalis*	13	3	16
* Escherichia coli*	12	1	13
* Klebsiella pneumoniae*	7	2	9
* Klebsiella oxytoca*	7	0	7
* Serratia marcescens*	5	0	5
* Enterobacter cloacae*	5	0	5
* Pseudomonas aeruginosa*	1	2	3
* Enterobacter aerogenes*	1	0	1
* Proteus mirabilis*	1	0	1
* Morganella morganii*	1	0	1
*Staphylococcus aureus* (methicillin-susceptible)	1	0	1
*Raoultella planticola*	1	0	1
Polymicrobial	7	2	9
UTI: did not meet diagnostic criteria			
* E. faecalis*	4	2	6
* E. coli*	3	0	3
* Citrobacter koseri*	1	1	2
* E. cloacae*	2	0	2
* K. pneumoniae*	0	2	2
* E. aerogenes*	1	0	1
* Pseudomonas spp*.	1	0	1
Polymicrobial	2	0	2
Culture not obtained	1	1	2
Antibiotic therapy:			
Intravenous:	40	6	46 (49%)
Gentamicin	24	5	29
Ampicillin	13	1	14
Cefazolin	5	0	5
Piperacillin/tazobactam	1	2	3
Ampicillin/sulbactam	2	0	2
Tobramycin	1	1	2
Other^a^	2	0	2
Enteral:	8	5	13 (14%)
Amoxicillin	2	4	6
Amoxicillin-clavulanate	3	0	3
Cephalexin	2	0	2
Trimethoprim-sulfamethoxazole, cephalexin	1	1	2
Intravenous to oral antibiotic therapy:	29	5	34 (37%)
Day (median, IQR) of therapy when changed from intravenous to enteral antibiotic	3(3–4)	3(3–3)	
Day 2	6	1	
Day 3	16	3	
Day 4	6	1	
Day 5	1	0	

^a^Ceftazidime, cefotaxime, cefepime, metronidazole.

*UTI* urinary tract infection, *IQR* interquartile range.

Four infants died following the UTI diagnosis; 1 of them died in the subsequent 7 days after stopping antibiotic therapy (Supplementary Table 1). The infant had an atrioventricular canal and died of complications of prematurity with bronchopulmonary dysplasia and pulmonary hypertension. None of the deaths were associated with recurrence of a UTI.

## Discussion

During the study period in this single-center study involving 7 NICUs, adherence to diagnostic criteria for uncomplicated UTI was 77% (72/93) with a median (IQR) length of antibiotic therapy of 5 (5–6) days (Table 4). The 5-day treatment course was associated with a 1% (1/91) failure rate defined as re-initiation of antibiotic therapy within 7 days after its discontinuation for a UTI due to the same organism. No infant death was associated with a UTI diagnosis (Table 4 and Supplementary Table 1).

The diagnosis of UTI among high-risk infants in the NICU is not well standardized and often dependent on the opinion of the neonatologist caring for the infant [15]. Efforts at standardization of diagnosis have shown improvement in guideline adherence, but there remains uncertainty when there is any bacterial growth on urine cultures [16]. In addition, pyuria is often absent even with significant bacterial growth on urine culture [17]. Collection of urine among high-risk infants in the NICU, especially uncircumcised males with unretractable penile foreskin, is hampered by use of collection methods that are subject to contamination. With these issues in mind, we developed a guideline mostly based on the diagnostic criteria set forth by the AAP UTI clinical practice guideline for older infants and children (Table 1). Despite education that was provided to all health care professionals with prospective auditing and feedback, 23% of UTI episodes did not meet our diagnostic criteria.

Short courses (3–7 days) of antibiotic therapy for UTI have been used among adult patients, but longer durations (7–14 days) are recommended by the AAP UTI guidelines in infants and children 2–24 months of age [8, 18]. A recent multicenter, randomized, placebo-controlled, noninferiority SCOUT study compared 5 vs. 10 days of antibiotic therapy for children aged 2 months to 10 years [19]. Among children assigned to 10 days of antibiotic treatment, 0.6% (2/328) had a treatment failure, defined as a symptomatic UTI at or before the first follow-up visit (day 11–14), compared with 4.2% (14/336) in the 5-day treatment group. The study failed to show that short course therapy was noninferior to a longer course, but since treatment failure occurred infrequently, the authors concluded that a short course could be a reasonable option for children exhibiting clinical improvement after 5 days of antibiotic treatment. In contrast, in a multicenter, randomized, Italian study of children aged 3 months to 5 years with noncomplicated febrile UTI, a 5-day course was noninferior to 10 days of oral amoxicillin-clavulanate [20]. The recurrence rate of UTI within 30 days after completion of therapy was 2.8% (2/72) in the short group and 14.3% (10/70) in the 10-day treatment group.

In a pragmatic, open-label, multicenter, Danish study of children aged 3 months to 12 years with febrile UTI, individualized treatment duration (median [IQR] 5.3 [4.8–6.5] days) had an increased risk of recurrent UTI within 28 days than those who received a standard 10-day treatment (11% vs. 6%, respectively) [21]. However, children in the individualized treatment group had reduced antibiotic use and fewer adverse event days. Similar data are not available for infants with UTI in the NICU. In a single center retrospective evaluation of 86 NICU infants, there was no difference in UTI recurrence between ≤7 days (27%, 7/26) versus >7 days (32%, 19/60) of appropriate antibiotic therapy [22]. In a subgroup analysis comparing <7 days vs. 7 days, infants who received short-course treatment had a higher incidence of recurrent UTIs, 5/10 (50%) vs. 1/11 (9%), respectively (*p* = 0.03).

As treatment duration for UTI among infants in the NICU is based on sparse evidence, and short-course antibiotic therapy for UTI appeared to be effective in some children and adults, our NEO-ASP recommended a 5-day antibiotic course with a time-out for treatment of “blood culture-negative” UTI [23, 24]. In addition, there is growing concern that prolonged or excessive antibiotic therapy may lead to perturbations in the infant’s bacterial microbiome with resultant adverse consequences [25–28, 29]. Prolonged antibiotic exposure in preterm infants has been associated with bronchopulmonary dysplasia, necrotizing enterocolitis, late-onset sepsis, invasive candidiasis, retinopathy of prematurity, neurodevelopmental impairment, and death, as well as emergence of multi-drug resistant organisms [29, 30]. Importantly, each additional day of antibiotic therapy increases the risk of adverse outcomes in infants ≤32 weeks’ gestation or birth weight ≤1500 grams after controlling for initial severity of illness [26, 31, 32]. In full term infants, prolonged therapy has been associated with delayed initiation of breastfeeding, obesity, recurrent wheezing, gastrointestinal disorders, as well as autism [29]. Among all infants, concern exists for potential toxicity such as hearing loss and renal dysfunction. For these reasons, and our past experience with 5 days of antibiotic therapy for pneumonia [33, 34] and “culture-negative” sepsis [34, 35], and 7 days for uncomplicated Gram-negative bloodstream infections in NICU infants [36], led to our decision for a shorter than usually recommended antibiotic course for non-bacteremic UTI with a time-out set at 5 days to decide whether to prolong the therapy. Although not fully comparable, the overall failure rate of 1% for short course antibiotic treatment in our study was similar to that seen in randomized studies in older children. In addition, a short course treatment duration appeared safe as there was no infection-related mortality associated with a recurrent UTI.

Adherence to the 5-day treatment duration for UTI was excellent, with the median duration of antibiotic therapy being 5 days. Although most (46/93, 49%; Table 4) of the antibiotic courses were provided intravenously, 14% (13/93) of UTI episodes were treated exclusively with enteral antibiotics. An additional 37% (34/93) of antibiotic courses were transitioned from an intravenous to an enteral route.

Limitations of the study include the relatively small study population as well as its observational nature that documented adherence to diagnostic and treatment following implementation of a NEO-ASP UTI guideline. The input from the NEO-ASP during the study period informed practice and was a driving force responsible for its implementation and adherence. Institutions without a strong ASP presence may have limitations in the generalizability of this guideline, especially with performance of the 5-day time-out. The diagnosis of UTI remained problematic as only 77% (72/93) of treated episodes of UTI were based on the guideline’s diagnostic criteria [37–41]. Nevertheless, the current design does give the study “real world” applicability. It is possible that not all eligible infants were identified during the study period. Since clinical pharmacists did not attend patient rounds on weekends and holidays, some infants with a treated UTI may not have been identified, although this was unlikely given the duration of antibiotic therapy. Cases where diagnostic criteria for UTI were met but the infant did not receive antimicrobial treatment, were not evaluated. Data on male circumcision also was not collected, although this elective procedure is not usually performed until the infant nears discharge to home. In addition, all of the 7 NICUs that participated in the study belonged to the same Nationwide Children’s Hospital Neonatal Network, and therefore, the efficacy and safety of the shortened course of antibiotic therapy should be studied in randomized clinical trials involving diverse demographic and geographic regions.

In conclusion, in our NICU network, there was moderate adherence (77%) to the NEO-ASP’s diagnostic criteria for UTI and excellent adherence to a 5-day definitive antibiotic treatment course for nonbacteremic UTI. No safety concerns were identified in this cohort, as only 1% of UTI episodes relapsed in the 7 days following completion of a 5-day median antibiotic treatment course. However, additional studies are needed that enroll larger study populations across other demographic and geographic centers. Ongoing education, prospective auditing and feedback, and collaboration among neonatal subspecialties helped inform antimicrobial prescribing in the NICU and allowed the successful incorporation of a short antibiotic course with “time-out” into our NEO-ASP bundle.

## Supplementary information

Supplementary information

## Data Availability

The de-identified dataset generated from the study is available from the corresponding author on reasonable request and following approval by the Institutional Review Board of Nationwide Children’s Hospital.
