# Supplementary material for: Urinary tract infection in the neonatal intensive care unit
Source: J Perinatol. 2026 Apr 29;46(5):754–60. doi: 10.1038/s41372-026-02690-1 (PMC13190328; doi:10.1038/s41372-026-02690-1)
Supplement: Supplementary file 1 — Supplementary information [file 41372_2026_2690_MOESM1_ESM.docx]

**Table S1. Deaths of 4 infants following diagnosis of urinary tract infection**

| **Cause of Death** | **UTI: Organism** | **Day of Life of Last UTI Treatment** | **Postnatal Age at Death** | **Positive Culture at Time of Death** |
| --- | --- | --- | --- | --- |
| NEC totalis | *Escherichia coli* | 25 | 44 | No |
| Hypoxic brain injury | *E. coli* | 50 | 316 | No |
| Partial midgut volvulus/silo/ compartment syndrome | *Klebsiella aerogenes* | 106 | 149 | *Klebsiella oxytoca:* peritoneal fluid |
| Pulmonary hypertension, atrioventricular canal, BPD | *Enterococcus faecalis* | 248 | 254 | *Serratia marcescens*: endotracheal tube |

UTI, urinary tract infection; BPD, bronchopulmonary dysplasia
